# How stressful was the COVID-19 pandemic for residents specializing in family practice?. A study of stressors and psychological well-being of physicians in further training specializing in family practice (GP trainees) within a pandemic context

**DOI:** 10.1186/s12875-022-01921-6

**Published:** 2022-12-01

**Authors:** Anna-Maria von Oltersdorff-Kalettka, Janina Meinel, Karen Voigt, Thomas Mundt, Markus Bleckwenn, Antje Bergmann, Mandy Gottschall

**Affiliations:** 1 Technische Universität Dresden, Medizinische Fakultät C. G. Carus der TU Dresden, Bereich Allgemeinmedizin, MKIII UKD, Kompetenzzentrum Weiterbildung Allgemeinmedizin Sachsen, Fetscherstraße 74, 01307 Dresden, Germany; 2 Universität Leipzig, Medizinische Fakultät der Universität Leipzig, Selbstständige Abteilung für Allgemeinmedizin, Kompetenzzentrum Weiterbildung Allgemeinmedizin Sachsen, Philipp-Rosenthal-Str. 55, 04103 Leipzig, Germany

**Keywords:** Coronavirus pandemic, Physicians in continuing education, Residents specializing in family practice (GP trainees), Psychological stress during a pandemic, resident stress, healthcare worker stress

## Abstract

**Background:**

The coronavirus pandemic poses many challenges for medical personnel. During the first phase of the pandemic, psychological stress became increasingly apparent. This was a complex and difficult situation, especially for physician residents specializing in family practice (GP trainees), who were not yet able to draw on years of practical experience. In this context, the Kompetenzzentrum Weiterbildung Allgemeinmedizin Sachsen (Competence Center for Continuing Education in General Medicine Saxony) (KWA^Sa^) developed a survey on how to deal with the concerns and challenges perceived at the time. The purpose of the study was to obtain information on psychological well-being in the pandemic context, as well as on expectations, fears, and protective measures in everyday work. The aim was to identify stress factors for general practice (GP) trainees during a pandemic situation to be able to consider the support needs in the design of future residency training programs, especially for GP trainees.

**Methods:**

An online questionnaire was distributed from May 5, 2020 to June 4, 2020 among GP trainees enrolled in KWA^Sa^ since 2018. The questionnaire consisted of standardized items, which were evaluated descriptively, and open-ended items with free-text answers, which were evaluated according to the principle of qualitative content analysis.

**Results:**

The results show the relevance of the topic as 61% of respondents indicated that they were concerned about the coronavirus. Most GP trainees also gave an affirmative response regarding emotional challenges. In this context, various stressors could be identified within both the professional and personal environments. There were four particularly salient factors: (1) the fear of infecting one’s family as well as patients with the SARS-CoV-2 virus; (2) missing or insufficiently existing protective measures; (3) an increased need for consultation due to unpredictable patient behavior as well as uncertainties in patient treatment; and (4) communication difficulties within the collegial environment.

**Conclusions:**

The study aimed to identify the support needs of GP trainees in crisis situations such as the COVID-19 pandemic. The results of the survey can be used for the development of suitable continuing education programs for physicians in further training.

**Supplementary Information:**

The online version contains supplementary material available at 10.1186/s12875-022-01921-6.

## Background

Residents specializing in family practice (GP trainees) experienced complex and problematic situations in their training practices/clinics as a result of the COVID-19 pandemic. Working in primary care offices, which are considered the first point of contact for many people when they become ill, held a high potential for spread and personal infection with SARS-CoV-2 at the onset of the 2020 pandemic. In this context, many medical personnel reported psychological symptoms or disorders that were fostered due to stressors related to the COVID-19 pandemic [1]. Holton et al. highlighted significant negative effects of the COVID-19 pandemic on the psychological well-being of clinical professionals [2]. Effects reported were stress, anxiety, depressive symptoms [3, 4]. Zerbini et al. also reported more stress, exhaustion, and depressed mood among medical staff, even independent of regular Covid-19 contact. The most common causes of stress were job overload and uncertainty about the future [5].

Studies have indicated that the number of positive COVID-19 cases in an area may be less important than the evaluation of the situation, a phenomenon which is related to social cognitive processes [1, 5]. This is, according to the view of a transactional understanding of stress (theory according to Lazarus & Folkman [6]), in which especially dangerous, delicate or very challenging transactions between the individuals and their environment are evaluated as stressful, decisive for the occurrence and occurrence of psychological symptoms [7]. In this context, the perception of stress was influenced by a variety of stressors that could increase the risk for symptoms and thereby reduce the quality of life [4]. Stress factors such as worries about one’s own health and that of family members played an important role in stress perception [8]. In this context, work-family conflict may have a strong negative impact on psychological well-being [9]. It can be assumed that the Corona pandemic favored the work-family conflict, since working as a physician in direct contact with the Corona virus also exposed the family to a higher and uncontrollable risk.

Knowing that pre-existing conditions increased the risk of a life-threatening course of COVID-19 may have acted as an additional stress factor among affected medical personnel [7]. In addition, a certain degree of discomfort, e.g., due to the lack or insufficiency of protective clothing/materials, as well as the care and treatment of (unstable) patients, were also circumstances that had a negative impact on the psychological well-being of medical personnel [10]. Another condition was job satisfaction and well-being at work [11].

Studies have found a link between the Corona pandemic and depression with effects within the workplace. For example, Obrenovic et al. analyzed using a cross-sectional study the impact of the Corona pandemic on depression and consequently decreasing job security in the United States, in their study on “The threat of COVID-19 and job insecurity impact on depression and anxiety” [12]. According to the results, there is a positive and highly significant impact of job insecurity on depression. Job insecurity thus leads to depression and to anxiety, according to Obrenovic et al. Both symptoms can be traced back to the Corona pandemic in the past year. Particularly discouraging in this regard had been the lack of knowledge, the inability to make reliable predictions, and the lack of coherent and accurate information about COVID-19 [12]. These findings could be noted or confirmed internationally. Khudaykulov et al. conducted a very similar study in Jiangsu province in China [13]. Again, the results show a significant association between the pandemic and economic deterioration with consequences on job insecurity and on psychological well-being, especially anxiety and depression [13]. Further extensive empirical research confirms these findings and demonstrates the stress and depression caused by job insecurity during COVID-19 [14–18].

Determinants of well-being include autonomy, control, mastery of the environment, social connectedness, self-efficacy, and a meaningful existence [19, 20]. In the context of many new situations caused by the COVID-19 pandemic, Brose et al. found that especially untrained or inexperienced staff showed a higher risk for the development of psychological symptoms [7].

This correlation also seems conclusive for physicians in training, because as untrained or inexperienced employees, they have not yet developed the previously mentioned determinants of well-being (autonomy, control, mastery of the environment, social connectedness, self-efficacy) to the same extent as physicians with many years of experience and a well-integrated work structure within their lives.

The COVID-19 pandemic had a negative impact on self-confidence at work [20]. Since GP trainees do not yet have years of experience as practicing physicians, this can also be hypothesized to occur for them. In order to counter such challenging situations and to reduce psychological strain and stress, increased social support should be made possible [21].

According to Guberina et al. the workplace acts as a buffer against fear, panic and anxiety. If this buffer falls away during a critical time, such as the Corona pandemic, the workplace is perceived as a threat and becomes a source of psychological stress [20]. This means that not only the Corona virus itself and the increased occurrence of the virus in medical facilities can lead to psychological stress, but additionally the perceived stability or instability at the place of work. Therefore, especially in a crisis, behavior on the part of managers/professional support persons plays a special role [20]. The provision of critical resources and positive incentives, such as orientation, training, motivation, psychological and social support, guidance, awards, and praise are indispensable for positive well-being. If these aspects are not adequately met, an increase in psychological unwellness could results [5, 20].

These supportive aspects play a particularly important role for physicians in further training, as they are still inexperienced personnel and thus do not yet have their own sufficiently large repertoire of points of orientation to counter such a crisis due to their limited work experience. As a result, physicians in further training are at a higher risk of developing psychological symptoms than physicians and medical staff with years of experience. In Germany, among others, the trainers and competence centers have the task of providing these positive impulses. For physicians in further training, they are the immediate reference persons and points of contact for uncertainties and questions.

So how was this Corona pandemic crisis and support for physicians in training perceived? Was the pandemic particularly stressful for this group? Were there factors that mitigated the stress of the Corona pandemic? Much of the literature to date refers generally to medical professionals and makes only limited distinctions between staff with many years of experience and those who are still in training. Differences between workplaces, such as intensive care units and ambulatory physician practices, have been examined to some extent, including between physicians and nurses. However, a specific look at physicians in training, who are particularly exposed to more uncertainties due to their training status and less experience in autonomy and dealing with patients, has been overlooked so far. This study will attempt to take a closer look at the conditions for this particular group and thus fill research gaps. The results presented here will show to what influence various stress factors have on well-being of trainees in general practice. In addition, it will also be discussed to what extent social support existed at the training site through instructors, mentors and contact persons within the residency training and at which points more support should be given in the future.

## Methods

### Design and target group

The KWASa is a state-supported institution for strengthening the continuing education of physicians in training during their residency. Within Germany, there are a total of 16 competence centers for general medicine. The KWASa is responsible for the federal state of Saxony in the east of Germany and consists of the two locations Dresden and Leipzig. As part of the assessment program of KWA^Sa^, a survey was conducted with the aim of identifying support needs of future GPs in a pandemic situation. For this purpose, an online questionnaire with Limesurvey was conducted during the first phase of the COVID-19 pandemic over a period of 4 weeks (from May 5, 2020 to June 4, 2020). The target group was the GP trainees enrolled in KWA^Sa^ since 2018 (*n* = 316, ca. 150 of whom are regular active members). A total of 73 GP trainees participated in the survey [22]. .Thus, the study participants were composed of physicians in further training from the area of Saxony, mainly from the urban areas of Dresden and Leipzig.

The study was divided into two different topics: emotional well-being of GP trainees, which is the topic of this article, and infomation level about COVID-19 of GP trainees, which has been published elsewhere [22].

### Recruitment and ethics

The link to the questionnaire was sent to the members registered in KWA^Sa^ by e-mail. Before the survey was started, written information on data protection and an electronic declaration of consent were obtained. Only after confirmation of the data protection conditions could the survey be started. Participation was voluntary, anonymous and the questionnaire could be cancelled at any time. Answers already given could be deleted at any time during the survey [22].

### Measurement tools

In addition to questions on sociodemographics, the questionnaire consisted of items assessing individual psychological well-being and stress characteristics due to the pandemic situation. Specifically, the following topics were queried: (1) fears in everyday work related to the COVID-19 pandemic; (2) worries and stresses caused by the pandemic situation; (3) evaluations of the protective measures implemented; and (4) feelings of support at the training site [22].

The survey was designed using a mixed-methods approach. Quantitative survey techniques and qualitative survey techniques both were used. In this study, it is a matter of methodological triangulation, or more precisely, embedded design: this design means that either quantitative or qualitative method predominates and the respective other method is used in a complementary manner to answer sub-questions already during data collection. In our study, the quantitative design predominated, while the qualitative design was used as a supplement to support the standardized surveys with individual statements. The quantitative design consisted of various standardized items. In addition to multiple-choice questions and yes/no questions, various likert scales were used to answer some items (depending on the question type). In addition to the standardized questions, some open-ended questions were used in the qualitative design. The goal of the open-ended questions was to gain insights beyond the specific research subject and standardized results. A total of seven open-ended questions related to stress and mental health were used. Due to the rapid pace of the Corona pandemic and lack of time resources, we did not use face-to-face interviews and asked the open-ended questions in writing. The specific open-ended questions can be found in Table 2.

Due to the current topic, there were further studies of other research networks at the time of the survey, which referred to other, but situationally similar target groups. To compare some results with the study results of other target groups, 3 items from already existing projects of other research networks were used for the topic “stressors and psychological well-being” of our study. This also had the advantage that the existing items had already been tested. On the one hand, items from the research project “COVI-Prim - Accompanying monitoring of primary care in family practices during the COVID-19 pandemic” of the Institute of General Medicine of the Goethe University Frankfurt am Main were used. In this project, the challenges that family physicians face during this pandemic and how they deal with them were recorded and analyzed. The target group here was family physicians. Two items from this study were used for our study. Second, an item from the study survey of the Applied Medical Psychology and Medical Sociology Research Group of the Department of Psychosocial Medicine and Developmental Neurosciences of the Carl Gustav Carus University Hospital at the Technical University of Dresden were used. The study analyzed how psychological well-being develops during such a pandemic and which factors affect it. Here, the target group was medical students. By using the items from these two research projects, better comparisons can be made between physicians in training and general practitioners or students. The specific items used and references to the research groups can be found in the Additional file 1: Appendix under “Appendix B - Items used from other projects”.

The questionnaire was tested by a cognitive pretest procedure. Since the quality of the collected data depends primarily on the comprehensibility of the questionnaire design, the cognitive pretest is a particularly suitable test method, since the response process of the respondents is actively scrutinized and examined for comprehension problems. This approach attempts to gain insights into the cognitive processes involved in answering questionnaires [23]. Cognitive pretesting is intended to bring out the non-functional parts or design flaws of the survey instrument, such as question problems, in order to counteract the unintended effects and to be able to improve the questionnaire. Various techniques are used in cognitive pretesting. The most important and most used cognitive techniques are probing, confidence rating, paraphrasing, card sorting, think-aloud and response latency [23].

In this study, card sorting was not used due to the corona pandemic situation. The methods used were:

- Comprehension-Probing: Inquiry meaning or their understanding about certain terms or word groups, inquiry follows immediately after answering the question
- Categoryselection-Probing: Inquiries about the choice of answer categories
- General-Probing: direct questioning about problems in answering the questionnaire
- ConfidenceRating: Questioning subjectively assumed reliability of an answer (certainty of answer) (How certain are you about your answer?)
- Paraphrasing: Read question aloud - reproduce content in own words
- Think-Aloud: Prompt to speak out loud thoughts during reflections

A test design was created in preparation for the test. For this purpose, the research team selected items that might be difficult or problematic and assigned them to different tests according to the methodological guidelines. The test design can be found in the Additional file 1: Appendix under *“Appendix A - Test design: cognitive pretest KWA*^*Sa*^ *survey: how stressful was the COVID-19 pandemic for family practice residents?”*

The cognitive test was conducted using this test design by means of three observational interviews. These lasted between 1 and 1.5 hours each. The interviews were recorded auditorily. Subsequently, a transcript and an observation protocol were created, which were qualitatively evaluated. Based on the qualitative evaluations, problems in the questionnaire were subsequently highlighted and rectified. Both the preparation of the test as well as the implementation and evaluations were carried out by a social scientist who was familiar with and experienced in cognitive pretesting.

In addition to the very time-consuming cognitive pretesting, the questionnaire was reviewed by experienced scientific employees of the Department of General Medicine at the University Hospital of the Technical University of Dresden and colleagues of the research team.

### Analysis methods

Subsequent to the survey, data analysis of the standardized survey sections was carried out by means of the csv values read out and using Excel and SPSS Statistics 27 (IBM). First, descriptive statistics were performed. Then, the open-ended questions were analyzed using the principle of qualitative content analysis according to Mayring [24] using the software MAXQDA (VERBI).

In the following, the qualitative analysis method of the free-text responses will be explained in more detail. As described above, seven open-ended questions were asked in the survey, in which participants had the opportunity to describe their feelings and thoughts as well as motivations and backgrounds on topics related to stress caused by the Corona pandemic by means of free-text answers (Table 2). In preparation for the asessment, the responses were recorded and sorted within a transcript. Since these were written rather than oral interviews, the responses could be transcribed directly and verbatim. The transcript can be found in the Additional file 1 Appendix under *“Appendix C - Transcripts of the open questions”.*

Since many of the answers were very detailed, qualitative content analysis according to Mayring was selected for more detailed analysis. This analysis method is a recognized method for qualitative data evaluation in Germany and originates from the field of empirical social research. The aim of the method is the organization and structuring of qualitative, i.e. latent and manifest data in the form of transcripts, observation protocols, video or image recordings [24]. Content analysis according to Mayring provides for three different methods of analysis: Summary, Explication, and Structuring. For this research project the summarizing content analysis was chosen, which is useful if the content level of the data is to be analyzed. Here, the data material was structured according to certain criteria by means of an inductive coding procedure. This means that the transcripts are first paraphrased and then bundled into categories [24].

MAXQDA was used as a tool for inductive categorization. This is a highly recognized software for computer-assisted qualitative data and text analysis, which facilitates the technical procedure and written recording of the coding.

Based on the qualitative content analysis, a total of nine categories with a total of 69 subcategories were identified in relation to the stresses and the handling of the Covid 19 pandemic. The elaborated code system can be found in the Additional file 1: Appendix under “Appendix D - Code system of qualitative content analysis according to Mayring”. The results of the content analysis are presented in the following chapters.

## Results

### Sample description and response behavior

Before presenting the interpretation of the results, we will take a look at the sample description and the response behavior of the participants.

A total of 73 physicians in continuing education participated in the survey. The survey took approximately 20 minutes to complete. To understand the participants’responses in the context of their current life situation, some socio-biographical data were collected, such as gender, age, workplace, etc. Table 1 shows the results of the sample description.

**Table 1:** Description of the survey participants, own illustration

Characteristic	% (n)	Characteristic	% (n)
**Gender:**		**Continuing Education Location**	
Female	78.1% (*n* = 57)	Hospital/Clinic	19.2% (*n* = 14)
Male	15.1% (*n* = 11)	Family practice	69.9% (*n* = 51)
Diverse	0.0% (*n* = 0)	Other	2.7% (*n* = 2)
Missing values	6.8% (n = 5)	Missing values	8.2% (*n* = 6)
**Age:**		**Care Level Clinic:**	
25–35 years	16.4% (*n* = 12)	Maximum care	5.5% (*n* = 4)
30–35 years	41.1% (*n* = 30)	Basic/regular care	10.9% (*n* = 8)
35–40 years	24.7% (*n* = 18)	Not applicable	2.7% (n = 2)
40–45 years	11.0% (n = 8)		
Missing values	6.8% (n = 5)	Missing values	–
**Continuing Education Year:**		**Practice Location:**	
1	5.5% (n = 4)	> 100,000 Inhabitants	35.6% (*n* = 26)
2	8.2% (n = 6)	20,000–100,000 Inhabitants	8.2% (*n* = 6)
3	15.1% (n = 11)	10,000–20,000 Inhabitants	9.6% (*n* = 7)
4	24.7% (n = 18)	5000–10,000 Inhabitants	8.2% (n = 6)
5	39.7% (*n* = 29)	< 5000 Inhabitants	8.2% (n = 6)
Missing values	6.8% (n = 5)	Missing values	–

In addition, the response behavior of the open-ended questions will now be discussed. As described in the methodology, in addition to the standardized questions, some open questions were also asked. Here the participants could describe their answers in a free text field. It can be noted that this type of response option was very well used. Table 2 shows the number of responses per open question.

**Table 2:** Number of answers of open questions, own illustration

Open question	Number of responses (Response rate in %)
Please briefly describe what the challenges were.	30 (41%)
Please briefly describe how you solved the challenges.	31 (42%)
Everyone reacts differently to such a pandemic, with different perceptions and ideas. Please briefly describe why you are concerned or not concerned about contracting COVID-19 yourself.	42 (57%)
What other measures have you implemented that have not yet been mentioned?	21 (29%)
What measures are proving effective/would you like to maintain after “normalization”? Please briefly justify your answer.	26 (36%)
What other measures would be useful in your eyes?	18 (25%)
Do you have any other comments on this topic?	10 (14%)

Within the responses to the open-ended items, the comprehensiveness and amount of information were a little mixed. A very large number of answers consisting of at least two longer sentences or bullet points can be found, as well as a very large number of answers consisting of four or more longer sentences or bullet points. Very short answers, consisting of one short sentence or one short bullet point, appear less frequently. Since the open-ended question types were items embedded in the online questionnaire, the answers given are not comparable with an interview conducted face-to-face. However, considering this written form of open-ended responses, a surprising amount of very detailed information can be found. The participants showed a pronounced and response behavior in terms of content, gave detailed and substantial answers, which indicates the importance of the topic queried here. The participants felt heard and made extensive use of the open questions to express their opinions. The response rate for the individual items in relation to the total number of participants is also largely satisfactory. However, it must be mentioned that the response rate decreased towards the end of the questionnaire. In relation to the content and the abundance of the individual responses, however, the responses did not lose quality.

### Mental well-being during the coronavirus pandemic

The time during the first phase of the COVID-19 pandemic in spring 2020 was perceived as stressful by the majority of GP trainees. Let’s first take a look at the statistical analyses of the anxiety or stress caused by the Corona pandemic.

**Fig. 1:**
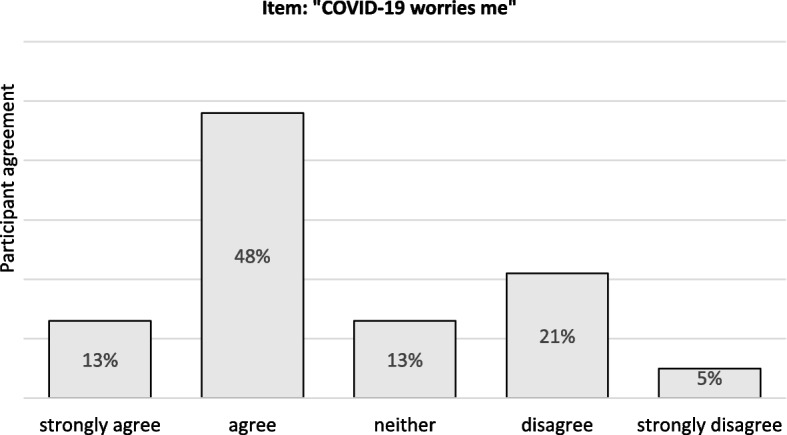
Perceived worries due to Covid-19, own illustration

To the statement “COVID-19 worries me,” 61% of respondents gave an affirmative response (13% indicated “strongly agree” and 48% indicated “somewhat agree”). A negative selection was made by 26% of GP trainees surveyed (21% “rather disagree” and 5% “strongly disagree”) and 13% abstained from a clear position with the answer option “neither”. Fig. 1 shows how pronounced the existing concerns were due to the Corona pandemic.

Concerns due to the corona pandemic were also evident within the open-ended free text responses. Worries and fears were openly addressed here:

“*Fear for the future for me and my children, fear, how the occupation can look in the future at all [...], fear that especially the children are to be socialized at a distance and masks, fear, for the education of my and all children. The own opportunities for further education are currently impossible for me. And much more*” (Additional file 1: Appendix C - Transcripts of the open questions; item no. 2; line 4–9).

“*Fear of self-infection, fear of unnoticed infection of my partner and my children by me. The patients’ severe psychological problems, which have clearly come to the fore in recent weeks, are also burdensome. The feeling of being abandoned by politics. Seeing how all around small businesses are struggling to survive and many of them will not survive the crisis, but of course the state wants to support the car industry*” (Additional file 1: Appendix C - Transcripts of the open questions; item no. 8; lines 23–28).

*“Concern about an outbreak, overburdening of the health- and economicsystem. Social isolation of the family, 1 school child of ours has to stay at home alone, every day. Worry of contagion, worry of chaos and anarchy.”* (Additional file 1: Appendix C - Transcripts of the open questions; item no. 22; lines 61–63).

These exemplary citations make it clear that during the Corona pandemic there were challenges perceived as stressful, such as those mentioned above: the political handling of the situation, anxiety about the impact of the Corona pandemic on the socialization of children, overburdening of the healthsystem and psychological problems of patients. Perceptions of emotional challenges were also statistically collected. When asked about emotional challenges triggered by the coronavirus pandemic, the majority of GP trainees surveyed also gave an affirmative response, with 50% of respondents indicating that they had experienced emotional challenges. In contrast, 27% of GP trainee respondents indicated that they had not experienced any emotional challenges, and 22% abstained from responding. Overall, it appeared that at the onset of the coronavirus pandemic, the majority of GP trainees questioned were concerned about the SARS-CoV-2 virus and experienced emotional challenges as a result of the pandemic.

### Stress aspects caused by the coronavirus pandemic

Let us now turn to elaborating on the specific emotional stresses and challenges caused by the Corona pandemic.

**Fig. 2:**
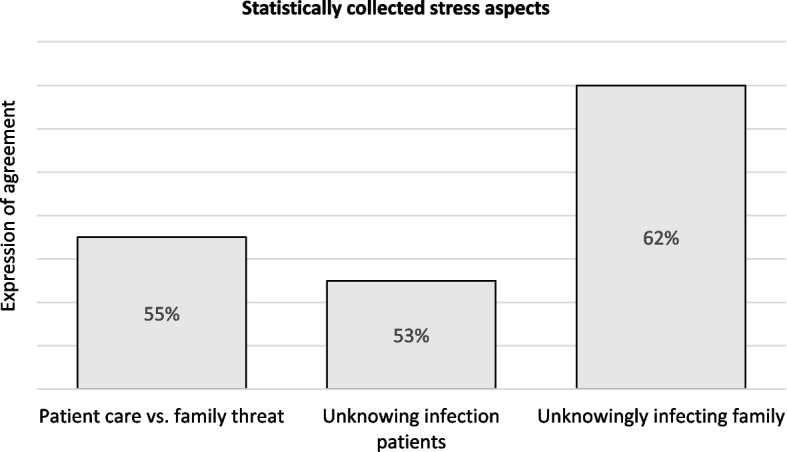
Expression of statistically surveyed stress aspects during the Corona pandemic

First, the statistical results of the survey showed that the existing worries related to the SARS-CoV-2 virus were mainly in the professional environment. Of the general practice trainees surveyed, 71% said that the work environment was a greater source of stress than the home environment. In this context, standardized questionnaires initially showed that about half of the GP trainees surveyed felt burdened by the prospect of unknowing infection due to working in a GP practice: 55% of the GP trainees surveyed felt burdened by the dilemma of wanting to provide good care for patients on the one hand and not wanting to endanger their families by working in a GP practice and the associated potential for increased risk of COVID-19 infection on the other. Of the trainees interviewed, 53% felt burdened by the possibility of unknowingly infecting patients. Feeling burdened by the increased potential to unknowingly infect family was reported by 62% of GP trainees surveyed. Fig. 2 shows the expression of these three aspects of stress during the Corona pandemic within the surveyed group of general practice trainees:

An affirmative response (strongly agree to somewhat agree) was given by 50% of GP trainees to the item “I feel I have little control over whether or not I contract COVID-19.”

In addition, the results of open-ended questions (using free-text responses) revealed further aspects of stress in the professional and private environment (Table 3).

**Table 3:** Stress factors and possible solutions - open question items, own illustration

Stress factors in the work environment	Stress factors within the personal environment	Solution approaches for coping with the stress factors
Collegial disagreements (n = 3)	Burden of social distancing (n = 5)	Establish strict organizational plans (n = 4)
Uncontrollable patient behavior (n = 6)	Fear of falling ill/infection in the family (*n* = 23)	Strict working time management (n = 6)
Increased effort per patient (*n* = 8)	Fear of own infection (n = 6)	Optimization Patient Care (n = 8)
Uncertainty in patient care (*n* = 9)	Sense of insecurity regarding the (private) future (n = 3)	Compliance with the rules of conduct (n = 6)
Lack of protective equipment (n = 7)	Difficulties in organizing everyday (family) life (*n* = 5)	Communication / exchange (*n* = 10)
Too few or contradictory (protective) instructions for behavior on the job (n = 9)	Political/media situation as a burden (*n* = 9)	Help from trainers and colleagues (n = 5)
(Loss) fear for the future of the profession (n = 3)	Fear of psychological effects – family (*n* = 2)	Independent reconnaissance (n = 5)
Fear of patient infection and consequences (n = 3)	Behavioral insecurities (n = 3)	Childcare (n = 2)
		Self-protection / self-care (n = 2)
		Production of own protective equipment (n = 2)

Within the qualitative evaluation of the free-text answers, various stress factors could be identified in relation to both the working and the private context. Table 3 shows in columns 1 and 2 the identified stress factors of both environments.

In the professional environment (column 1), aspects that make patient treatment more difficult (Uncontrollable patient behavior, Increased effort per patient, Uncertainty in patient care) are particularly noticeable. These aspects appear to be the most important stress factors within the field of work. The following citations are intended to provide some insight into the perceived stress:

*“Panicked and overwhelmed patients despairing between homeschooling, toddler care, and job in three-shift system”* (Additional file 1: Appendix C - Transcripts of the open questions; item no. 17; lines 50–51).

“*[…] in addition I have the feeling that I cannot help my patients, most of whom are mentally ill, as much as I would like to at the moment due to the considerable restrictions in their daily lives, since aftercare services, support groups and the like do not take place for an indefinite period of time, or only to a very limited extent.”* (Additional file 1: Appendix C - Transcripts of the open questions; item no. 23; lines 64–68).

*“[…] The patients’ severe psychological problems, which have clearly come to the fore in recent weeks, are also a burden. […]”* (Additional file 1: Appendix C - Transcripts of the open questions; item no. 8; lines 24).

*“[…] patients who visit the practice with suspected cases despite all indications and only become concrete in the consulting room and want to be tested […]”* (Additional file 1: Appendix C - Transcripts of the open questions; item no. 3; lines 13–14).

In addition, a lack of protective equipment and too few instructions for procedures also play a role in increased stress and strain.

*“[…] Endangerment of high-risk patients due to lack of protective equipment […]”* (Additional file 1: Appendix C - Transcripts of the open questions; item no. 13; lines 43).

*“[…] In some cases, the clinic management was also overtaxed, which led to uncertainty and a lack of a clear line.”* (Additional file 1: Appendix C - Transcripts of the open questions; item no. 28; lines 75–76).

Within the private environment, it is above all the fear of infecting the family, the own infection as well as the unclear political situation within the Corona pandemic, aspects which promote emotional stress.

*“Above all, I am worried about infecting or endangering my children, my husband or our parents and my grandmother […]”* (Additional file 1: Appendix C - Transcripts of the open questions; item no. 63; lines 134–135).

*“it is not clear how severe the course would be for me and how much I would infect my family and they would suffer from it”* (Additional file 1: Appendix C - Transcripts of the open questions; item no. 74; lines 168–169).

Participants were also asked about successful approaches they had used to manage aspects of stress (see Table 3, column 3). Here, communication and optimization of patient care played the most important role

### Protective measures to reduce the feeling of insecurity

In order to be able to ensure safety in everyday professional life, some protective measures were recommended for GP practices at the beginning of the pandemic [25]. The results of the standardized survey of the actual use of the recommended protective measures showed a heterogeneous picture (Table 4).

**Table 4:** Protective measures used (as of May 2020), own illustration

Implementation of measures in %. Standardized queried		Other measures used - named within the open question ite
Sewn masks and nose protection	83%	Controlled spacing (e.g., by chair markings) (n = 9)
Cancellation of routine appointments	73%	Patient admission strongly controlled (n = 6)
Acrylic glass panes	62%	Pre-registration by telephone (n = 4)
Hand disinfection for patients	53%	Infectious disease waiting room (*n* = 4)
Cancellation of home visits	48%	Patients mask (n = 3)
Wearing gloves	48%	Strongly regulated pickup of medications or prescriptions (*n* = 3)
FFP2/3 mouth-nose protection	45%	Reduction of collegial gatherings (n = 2)
Consultation hours for infections	45%	Reduction of contact points (e.g., due to open doors - avoidance of door handles) (n = 2)
Adjustment of office hours	37%	Query of risk factors (n = 1)
Telemedicine	34%	Video consultation (n = 1)
Wearing face shields	30%	Increased patient education (n = 1)
Wearing protective gowns	26%	

A few physicians in further education even saw no solution or way out at all, which was especially shown by the open free text answers:

„*The problems cannot be solved, or they increasingly show already existing structural problems.”* (Additional file 1: Appendix C - Transcripts of the open questions; item no. 38; lines 91–92).

*“There is no solution. I felt like I was on the verge of a nervous breakdown and now I’m on vacation and trying to distract myself.”* (Additional file 1: Appendix C - Transcripts of the open questions; item no. 39; lines 93–94).

### Sense of support at the training site

**Fig. 3:**
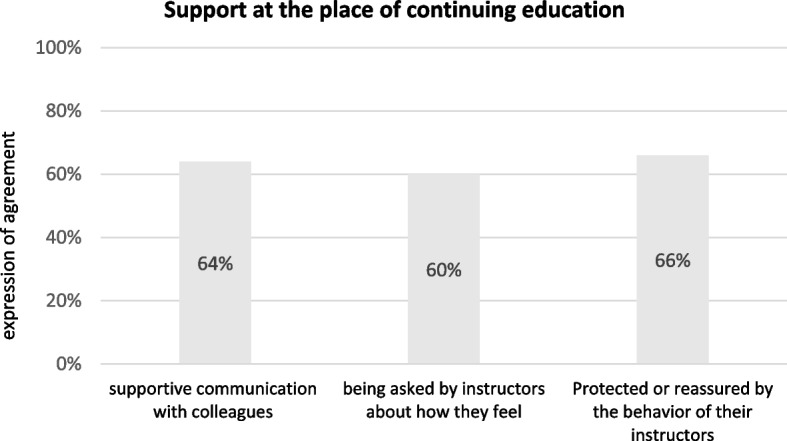
Support at the training site

Another aspect that can play a role in reducing uncertainties regarding the COVID-19 pandemic is “job satisfaction” - the situation directly at the workplace in connection with the cooperation with the respective continuing education instructor(s) and colleagues. The evaluations showed positive results at this point the majority of the GP trainees felt supported at their place of continuing education. Specifically, 64% of the respondents stated that there was mutually supportive communication with their colleagues during the first pandemic phase, 60% of the GP trainees surveyed were asked by their respective continuing educator(s) how they felt, and 66% felt protected or reassured by the behavior of the continuing educator(s). The results can also be traced in Fig. 3.

These data show that in about two-thirds of the cases, the mutual interaction within the continuing education location was able to convey a feeling of security and support during this very uncertain time.

However, despite some support from the training and continuing education site, as the results here showed, there was strong anxiety and worry about the pandemic period (see Fig. 1 and the open free text responses described above). Fear for family and major uncertainties/stresses in patient care seem to play the largest role, as shown above. Nevertheless, 1/3 of respondents did not feel safe or supported at the training site by the behavior of supervisors and caregivers. While this is not the majority, 1/3 of all respondents are a variable that cannot be ignored. Worry in the workplace was also confirmed in the open free text responses:

*“challenge in dealing with my boss, who perceived the corona crisis as of little importance and I have few options for action on my own.”* (Additional file 1: Appendix C - Transcripts of the open questions; item no. 4; lines 16–17).

*“In some cases, the clinic management was also overtaxed, which led to uncertainty and a lack of a clear line.”* (Additional file 1: Appendix C - Transcripts of the open questions; item no. 28; lines 75–76).

This shows the need for supportive communication in the workplace. The results of the standardized questions also confirm this. As a safety-giving measure, 49% of the GP trainees surveyed wished for more offers of emotional support (50% did not).

### Summary of results: stressors among physicians in training during the Corona pandemic

The above results indicate that the Corona pandemic had a negative effect on the well-being of physicians in training.

Sixty-one percent of GP trainees surveyed indicated that they were concerned about the coronavirus. Most of the GP trainees surveyed also gave an affirmative response regarding coronavirus-related emotional challenges. Regarding the emotional challenges experienced, various stress factors could be identified within both their professional and personal environments. These stressors have been presented so far in the chapters above and will now be summarized here in a final way to the most prominent characteristics. There were four stress factors that particularly stood out. These include:

(1) Anxiety/ fear: the fear of infection of the family as well as of the patients with the SARS-CoV-2 virus;

(2) low protective measures: lack of or insufficient protective measures (protective equipment and protective handling instructions);

(3) difficult patient care: an increased need for counseling due to unpredictable or uncontrollable patient behavior and uncertainty in patient care; and.

(4) insufficient social support: a need for more communication and experienced social support within the collegial environment and in the context of continuing education programs.

### Summary and discussion

How was the Corona pandemic crisis and support perceived by physicians in training? Was the pandemic particularly stressful for this group? Are there factors that may mitigate the stress of the Corona pandemic?

Overall, the results presented here confirm previous research on medical personnel with contacts to the SARS-CoV-2 virus. Concerns about one’s own health and that of others, insufficient social support, lack of information (regarding current guidelines, adequate treatment of COVID-19) as well as lack of protective equipment seemed to be conditions in all medical fields worldwide that increased the perception of stress and thus represent a risk for psychological well-being [4, 11, 24].

Symptoms resulting from the pandemic were already identified by Zerbini et al. These are increased stress, fatigue and depressive mood [5]. The psychological stresses are closely related to the workplace. Again, the results of this study confirm previous research literature by Obrenovic et al. and also Khudaykulov et al. [9, 12].

A lower sense of control and little mastery of the social environment are determinants of well-being according to Guberina et al. [20]. The results of this study show that a loss of control of patient behavior, especially impulsive and moody patients, and the feeling of being able to help the pateints little to not at all were very stressful for the physicians in training (stress factor 3). Thus, this study confirms that the determinants of well-being mentioned by Guberina et al. broke down during the Corona crisis, and psychological unwellness resulted as a consequence. The feeling of loss of control in patient care is still a poorly studied phenomenon in the literature. With regard to the group of physicians in training, who, as novices in the medical field, still have very little experience with such loss-of-control experiences, no knowledge exists to date. The results of this study show that, especially for newcomers to the profession, the aspect of loss of control and mastery of situations in the Corona pandemic had a particularly stressful effect.

Especially the connection between family and work can lead to stress and unhappiness, as Obrenovic et al. pointed out [9]. This was shown to be the main problem in the results of this study (stress factor 1). The fear of endangering the family through professional contact with Covid-19, the dilemma of caring for patients and thereby endangering the family higher as well as the poor compatibility of professional duties (increased working hours) with the care of children were the main responsible characteristics for psychological stress. Thus, the results confirm the theses of Obrenovic et al. Moreover, this characteristic turns out to be the main reason for stress during the Corona pandemic among physicians in training. The conflict between family and work due to the pandemic situation led to a higher stress than the virus itself, because the fear of one’s own infection for one’s own health played a smaller role than family security. This is particularly problematic for young physicians in continuing education, as they are often in the early stages of family time with their comparatively young years, unlike older colleagues.

Many studies indicate that job satisfaction played an important role in relation to stress during the coronavirus pandemic and that anxiety and stress are negatively related to job satisfaction [11–13]. The protective measures in place play an important role (stress factor 2). But Tracy et al. also pointed out that a lack of supportive social environment acts as a major stressor [26].

The study by Suryavanshi et al., which was conducted with a total of 197 healthcare professionals (doctors, nurses, doctors in training/internship) in India, also showed the relevance of work environment to the risk of combined depression and anxiety [4] In this context, Guberina et al. describe a very important factor: the place of work as a buffer in times of crisis [20]. Thus, a supportive, safe and satisfied feeling at the workplace seems to be an aspect that contributes significantly to the positive perception of the situation within the coronavirus pandemic and, consequently, to the sense of stress, which is why this point should be of particular concern for the centers of excellence and continuing education in their missions. The “buffer in times of crisis” is an aspect that is of particularly high importance for the competence centers, trainers and contact persons of physicians in continuing education (stress factor 4), since the competence centers and trainers carry a certain responsibility for the quality of this support.

Lack of communication and mutual support in the professional environment and in continuing education can bring the danger of strain, such as collegial disagreements with instructors here. On the other hand, good communication within the team was indicated as a solution strategy to improve the situation. This shows the importance of communication and support. The responses to the standardized items on the feeling of support also showed the relevance of positive communication and support from the continuing education program. Most of the physicians surveyed stated that they felt supported at the training site and protected by the behavior of the continuing education instructors. Nevertheless, for about one third of the GP trainees surveyed, there was no supportive environment.

Also, within the results of our study it can be stated that the communication and the feeling of support at the place of continuing education can have both mitigating and negative or stressful effects on the psychological well-being of the GP trainees. Therefore, it seems even more important to strengthen the education and training of GP trainees, for example by preparing trainers for crisis-specific and challenging communication.

As described in the literature, the number of positive COVID-19 cases in the area was less important than the evaluation of the situation [1; 5]. It should be possible for the professional environment of physicians in training (the competence evaluationcenters and trainers) to support this crisis situation in such a way that the evaluation of the situation is more positive and thus there are fewer burdens. As Brose et al. pointed out, support is especially important for untrained staff, as they are at higher risk for developing psychological symptoms [7]. The studies by Tracy et al. and Labrague and de los Santos also confirmed that untrained medical staff and lack of training contribute to an increase in feelings of stress [11] and 49% of the GP trainees surveyed in the present study also wished for more offers of support. Competence centers can and should therefore respond with support services specifically designed to address this issue, such as crisis-specific education and training. Initial studies have shown the success of such crisis-specific trainings: Khan & Kiani presented in their work on “Impact of multi-professional simulation-based training on perceptions of safety and preparedness among health workers caring for coronavirus disease” the success of first simulation trainings concerning the handling and treatment of COVID-19 patients [27]. By training typical procedures, such as in this case the testing of COVID-19 patients, blood sampling, cleaning and hygiene measures, etc., the perception of risks and the (treatment) preparedness of health care workers could be improved, and the feeling of safety increased [27].

Supportive aspects play a particularly important role for physicians in further training. Due to their limited professional experience, they do not yet have a sufficiently large repertoire of orientation points of their own to counter such a crisis. Physicians in further training therefore have a higher risk of developing psychological symptoms than physicians and medical staff with many years of experience. A specific look at physicians in postgraduate training, who are particularly exposed to many uncertainties due to their level of training and less experience in working independently and with patients, has not been strongly considered in previous research. This study shows that this group has special support needs and that there are also opportunities to better support physicians in continuing education in times of crisis.

### Strengths and weaknesses

A strength of this publication is that it presents data on stress factors experienced by GP trainees during a pandemic situation. Previous literature on the corona pandemic often differentiates between physicians and nurses or the different areas, such as the intensive care unit or corona outpatient clinic. However, a specific look at physicians in training is lacking. This study will advance research for this specific group. The stress factors can be considered in the future for support needs in residency education and training and can be used for the design of continuing education programs for GP trainees. In Germany, centers of excellence in general practice are still very young (the call for proposals started in 2017). Therefore, research that advances the work of the centers of excellence and trainers for physicians in continuing education in general practice is important.

A limitation is that the results presented here are attributable to a specific and small target group with a rather low response rate. KWA^Sa^ refers specifically to Saxony, a federal state in Germany. To generalize the results nationwide, further studies in other German states are necessary.

## Conclusions

GP trainees faced emotional and psychologically stressful challenges during the initial pandemic phase due to certain factors described here. A distinctive feature for the target group addressed in this study is that these GP trainees are still in the training phase and should therefore be provided with a specific level of support. At the same time, centers of excellence share responsibility for this support and can interpret the results to some extent as an evaluation of the quality of support provided by continuing education. The results show the importance of communication and support by the respective trainers. It is the task of the competence centers to support them in this respect and to teach the trainers how to deal with their GP trainees in a supportive way. However, crisis-specific training should also be included and implemented in continuing education. Within KWA^Sa^, we have already conducted seminars on topics related to the COVID-19 pandemic. We also want to contribute in the future to the communication of supportive measures for such uncertain experiences as a pandemic brings with it.

## Supplementary Information

**Additional file 1.**

## Data Availability

In principle, it is correct (also in terms of sustainability) that one can release data sets for subsequent use. This assumes, though, that they are fully anonymous, i.e. that there is no risk of exposing individuals. However, our data relate to a very specific group in Saxony in Germany. We collected sociobiographical data, such as age, gender, place of residence (city vs. country). With good research, the release of the dataset has removed the anonymity of some participants. This argues against a general release of the raw data. Contact: Anna-Maria von Oltersdorff-Kalettka, Dipl.-Soz., Phone: + 49 (0) 351 458 19647 mail: Anna-Maria.vonOltersdorff-Kalettka@uniklinikum-dresden.de, Competence center for further training in general medicine in Saxony KWA^Sa^ | Technical University of Dresden | University Hospital Carl Gustav Carus | Department of General Practice / Medical Clinic and Polyclinic III | Fetscherstr. 74 | 01307 Dresden, Germany.

## Abbreviations

KWA^Sa^: Competence Center for Continuing Education in General Medicine Saxony / Kompetenzzentrum Weiterbildung Allgemeinmedizin Sachsen
GP trainees: Residents in family practice
