# Supplementary material for: How stressful was the COVID-19 pandemic for residents specializing in family practice?. A study of stressors and psychological well-being of physicians in further training specializing in family practice (GP trainees) within a pandemic context
Source: BMC Prim Care. 2022 Dec 1;23:308. doi: 10.1186/s12875-022-01921-6 (PMC9713726; doi:10.1186/s12875-022-01921-6)
Supplement: Supplementary file 1 — Additional file 1. [file 12875_2022_1921_MOESM1_ESM.docx]

**How stressful was the COVID-19 pandemic for residents specializing in family practice?**

A study of stressors and psychological well-being of physicians in further training specializing in family practice (GP trainees) within a pandemic context.

Anna-Maria von Oltersdorff-Kalettka ^1^, Janina Meinel ^1^, Karen Voigt ^1^, Thomas Mundt ^2^, Markus Bleckwenn ^2^, Antje Bergmann ^1^, Mandy Gottschall ^1^

Project Manager:

Mandy Gottschall, Head of training division

Tel. + 49 (0) 351 458 19289
[Mandy.Gottschall@ukdd.de](mailto:Mandy.Gottschall@ukdd.de)

Competence center for further training in general medicine in Saxony KWA^Sa^ | Technical University of Dresden | University Hospital Carl Gustav Carus | Department of General Practice / Medical Clinic and Polyclinic III | Fetscherstr. 74 | 01307 Dresden, Germany

Lead author/ Corresponding author:

Anna-Maria von Oltersdorff-Kalettka, Dipl.-Soz.

Tel. + 49 (0) 351 458 19647
[Anna-Maria.vonOltersdorff-Kalettka@uniklinikum-dresden.de](mailto:Anna-Maria.vonOltersdorff-Kalettka@uniklinikum-dresden.de)

Competence center for further training in general medicine in Saxony KWA^Sa^ | Technical University of Dresden | University Hospital Carl Gustav Carus | Department of General Practice / Medical Clinic and Polyclinic III | Fetscherstr. 74 | 01307 Dresden, Germany

^1^Technische Universität Dresden, Medizinische Fakultät C. G. Carus der TU Dresden, Bereich Allgemeinmedizin, MK III UKD, Kompetenzzentrum Weiterbildung Allgemeinmedizin Sachsen, Fetscherstraße 74, 01307 Dresden

^2^Universität Leipzig, Medizinische Fakultät der Universität Leipzig, Selbstständige Abteilung für Allgemeinmedizin, Kompetenzzentrum Weiterbildung Allgemeinmedizin Sachsen, Philipp-Rosenthal-Str. 55, 04103 Leipzig

***Appendix***

Appendix A - Test design: cognitive pretest KWA^Sa^ survey: how stressful was the COVID-19 pandemic for family practice residents?

Techniques used:

- Comprehension-Probing: Inquiry meaning or their understanding about certain terms or word groups, inquiry follows immediately after answering the question
- Categoryselection-Probing: Inquiries about the choice of answer categories
- General-Probing: direct inquiry about problems in answering the questionnaire
- ConfidenceRating: Inquiry about subjectively assumed reliability of an answer (answer certainty) (How certain are you about your answer?)
- Paraphrasing: Read question aloud - reproduce content in own words
- Think-Aloud: Prompt to speak out loud thoughts during reflections

Items to be tested :

| Item-No. | Item | Potential problems | Tests |
| --- | --- | --- | --- |
| B1 | Please think about the last three weeks. Have you faced any emotional challenges in connection with the COVID 19 pandemic? challenges that you faced? | Emotional challenges - definition - when does a challenge begin? - possibly a term that first needs to be thought about for a long time | Comprehension-Probing  What does an emotional challenge mean to you? |
| B2 | Please briefly describe what challenges these were and how you solved them. | Item requires reflection - possible satisficing risk | Think -Aloud  Please read the question aloud and then try to speak your thoughts aloud throughout. |
| B3 | How do you generally deal with emotional stress?  (Multiple answers possible) | Multiple answers were criticized by colleague in feedback - prefer scaling? - are the items good to answer? | General-Probing  Do you find it difficult to select these items clearly? Do you have problems reading and answering the question? |
| B5 | Please think about how you feel about the current Corona-Pandemic. Which of the following statements apply to you?  7 items that require a longer reading time | Longer reading time possibly leads to satisficing  + scale can answer the questions well? | ConfidenceRating  How confident are you in your answers? |
| D1 | I feel like I have not much control over whether or not I contract  I am infected with COVID-19 or not. | Length + complexity - lots of strong terms in there - feeling, control, covid19, contagion. | Paraphrasing  Please paraphrase this statement in your own words  ConfidenceRating  How confident are you about your answer? |
| D1 | I am willing to take the risks involved to help COVID-19 patients. | Length + complexity 🡪 long nested sentence, long cognitive attention 🡪 possibly satisficing.  How much "thinking" willing to expend? | Think aloud  Read through the question and immediately after reading it, begin to speak your thoughts aloud as you ask the question. |
| D1 | I am sure that we can overcome COVID-19.  After COVID-19, our world will change. | Fuzzy formulations: what is meant by this? Can this therefore be answered at all? | Categoryselection-Probing  Why did you choose this answer? |
| D3 | Every person reacts differently to such a pandemic and has different ideas and perceptions. Please briefly describe why you are worried or not worried about contracting COVID-19 yourself. | Long and complex question + open question requires high cognitive attention - risk satisficing | General-Probing  Do you find this question complicated? Do you have problems reading and answering the question?  Or Think Aloud  Please read the question aloud and then try to speak your thoughts aloud throughout. |
| D5 | Now think about patient and professional management in your everyday professional life. Which of the following measures have been implemented in your work environment as a result of the Corona crisis? With regard to the following statements, please indicate the extent to which they apply to they apply to you. | ength and complexity 🡪 some colleagues in feedback were confused or criticized why we use the scale -- possibly question too long? Question is not remembered and then scale becomes confusing when answered | Paraphrasing  Bitterly try to reproduce the question in your own words |
| D6 | What other measures have you implemented that have not yet been mentioned? | open question 🡪 requires high cognitive attention and willingness to remember 🡪 risk satisficing  + prior knowledge: Proband may not know this | Think Aloud  Please read the question aloud and then try to speak your thoughts aloud throughout. |
| D7 | Which measures are proving successful/do you want to maintain after a "normalization"? Please give reasons for your answer briefly. | open question 🡪 requires very very high cognitive attention and willingness to reflect 🡪 risk satisficing | Think Aloud  Please read the question aloud and then try to speak your thoughts aloud throughout. |
| D8 | In my professional environment we are encouraged to exchange with colleagues in order to mutually | Long question + willingness to remember/reflect 🡪 risk satisficing | Categoryselection-Probing  Why did you choose this answer? |
| D8 | I feel protected/reassured by the behavior of my continuing education instructor. | High cognitive effort  Willingness to remember/reflect + can this be answered well?  Risk satisficing | ConfidenceRating  How confident are you in your choice? |
| E3 | Please think of a situation where you yourself are looking for current information on the COVID-19/SARS-CoV-2. Which provider do you seek information and how do you feel about the information provided by the respective providers? | Length and complexity | Paraphrasing  Bitterly try to reproduce the question in your own words |
| E3 | General Practitioners' Association, Robert Koch Institute (RKI), Association of Statutory Health Insurance Physicians (KV), DEGAM, Deximed  (German medical associations/institutions) | Prior knowledge - definition | Comprehension-Probing  Do you know all of the terms mentioned or can you clearly assign/ define the terms mentioned? |
| E7 | Where do you see potential to improve or expand information sharing and communication and support? | Open question risk satisficing | Think Aloud  Please read the question aloud and then try to speak your thoughts aloud throughout. |

Appendix B - Items used from other projects

Note: The research projects are conducted in Germany in German. The authors have translated the items into English independently

**project: "COVI-Prim - Accompanying monitoring of primary care in family practices during the COVID-19 pandemic" of the Institute for General Medicine of the Goethe University Frankfurt am Main**

Research Group Website: <https://www.allgemeinmedizin.uni-frankfurt.de/89698463/COVI_Prim>

**Item used:**

Please think about how you feel about the current Corona pandemic. Which of the following statements apply to you?

I am burdened by the fact that I want to care for my patients on the one hand, but also do not want to endanger my family.

I am afraid that I will infect a patient with COVID-19

I am worried that I could infect people I live with with COVID-19.

I am concerned that I may unknowingly infect my patients.

When I think about my patients who are infected with COVID-19, I feel helpless.

When dealing with patients infected with COVID-19, I am sometimes unsure if I am doing everything right.

My colleagues are afraid of infecting patients with COVID-19

Answer scale: Yes, rather Yes, rather No, no, no answer

**Item used:**

Please think about how you feel about the current Corona pandemic. Which of the following statements apply to you?

I have children to care for at home.

I have parents or grandparents to care for.

I have moved away from home so as not to endanger my family.

Answer scale: Yes, No, No answer

**Project: Study survey of the research group Applied Medical Psychology and Medical Sociology of the Department of Psychosocial Medicine and Developmental Neurosciences of the University Hospital Carl Gustav Carus at the Dresden University of Technology**

Research Group Website: <https://www.uniklinikum-dresden.de/de/das-klinikum/kliniken-polikliniken-institute/psm/angewandte-entwicklungsneurowissenschaften/medizinische-psychologie-und-medizinische-soziologie>

**Item used:**

With regard to the following statements, please indicate to what extent they apply to you:

I am afraid/have been afraid of contracting COVID-19.

I feel I have little control over whether or not I contract COVID-19.

I am afraid of infecting others with COVID-19.

My family and friends are afraid that they will contract COVID-19 from me.

I am willing to take the risks involved to help COVID-19 patients.

I am concerned about COVID-19.

I am confident that we can overcome COVID-19.

In the face of COVID-19, many things don't seem so important to me anymore.

After COVID-19, our world will change.

Appendix C - Transcripts of the open questions

Note: the answers were translated into English by the authors

| **Please briefly describe what the challenges were.** | |  |
| --- | --- | --- |
| Answer | Number | Line |
| - Low partly contradictory instructions for action  - lack of protective equipment  - many discussions with patients | 1 | 1  2  3 |
| - Fear for the future for me and my children, fear, how the occupation can look in the future at all (related to Covid19 measures and the general suspension of rights and data protection regulations of the patients and my own by Mr. Spahn (Minister of Health in Germany)) - fear that especially the children are to be socialized at a distance and masks, fear, for the education of my and all children. The own opportunities for further education are currently impossible for me. And much more | 2 | 4  5  6  7  8  9 |
| -Suspected cases with an open result, this will be the case more and more often, especially when it is felt, and the uncertainty then remains partly over holidays and the weekend, how to behave, enjoy your free time without being careless - it is a balancing act -patients who visit the practice with suspected cases despite all indications and only become concrete in the consulting room and want to be tested -the possibility of endangering/infecting friends and family | 3 | 10  11  12  13  14  15 |
| 1. challenge in dealing with my boss, who perceived the corona crisis as of little importance and I have few options for action on my own. 2. Many conversations around anxiety and panic in people, focus on mental disorders. | 4 | 16  17  18 |
| Rejection of protective measures by the public and by patients, irrational demonstrations and demands. | 5 | 19  20 |
| Fear of infecting family | 6 | 21 |
| Fear of not being able to combine childcare and work | 7 | 22 |
| Fear of self-infection, fear of unnoticed infection of my partner and my children by me. The patients' severe psychological problems, which have clearly come to the fore in recent weeks, are also a burden. The feeling of being abandoned by politics. Seeing how all around small businesses are struggling to survive and many of them will not survive the crisis, but of course the state wants to support the car industry. | 8 | 23  24  25  26  27  28 |
| Fears regarding the disease for my family, friends and me, missing contacts, annoyed by pseudo-scientists |  | 29  30 |
| Continuation of full-time medical work and emergency care of my own children - this was an emotional challenge for me. | 9 | 31  32 |
| Felt increased risk of infection, despite own immunosuppression little fear for self, but did for relatives from at-risk group | 10 | 33  34 |
| Homeschooling 100% childcare without daycare o grandparent support. | 11 | 35 |
| I was ridiculed, almost scorned by my boss for my attempt to implement all RKI rules on patients (distance, treating with mask, extra gown); she herself made no effort for 6 weeks to channel patient flow, cancel petty requests or the like. In doing so, she endangered me and my family. Only after 6 weeks I was "allowed" to treat with mask. Had already brought mine from the beginning and privately wore gloves during examination. | 12 | 36  37  38  39  40  41 |
| Isolation of newly admitted patients in nursing homes  Endangerment of high-risk patients due to lack of protective equipment and negligent behavior of other patients | 13 | 42  43  44 |
| no protective clothing too many psychologically and physically decompensated with patients aggressive, demanding patients | 14 | 45  46 |
| Reallocate child care Possible pending additional workload- did not materialize, but was not foreseen | 15 | 47  48 |
| Keep contact restrictions private | 16 | 49 |
| Panicked and overwhelmed patients despairing between homeschooling, toddler care, and job in three-shift system | 17 | 50  51 |
| Patients increasingly requested a corona smear even though the indication for it was not formally given. In this case, fear was clearly in the foreground. In most cases, these were patients who had also previously been noticed with psychosomatic clinical pictures. | 18 | 52  53  54  55 |
| Patients have a high need for information and assessment of the situation. | 19 | 56  57 |
| Patients stabilize by the compulsory measures at the edge of itself stood financially emotionally physically | 20 | 58  59 |
| patients full of fear to take them, to work without adequate protective clothing | 21 | 60 |
| Concern about an outbreak, overburdening of the health, and economic system. Social isolation of the family, 1 school child of ours has to stay at home alone, every day. Worry of contagion, worry of chaos and anarchy. | 22 | 61  62  63 |
| Social isolation as a person living alone, in addition I have the feeling that I cannot help my patients, most of whom are mentally ill, as much as I would like to at the moment due to the considerable restrictions in their daily lives, since aftercare services, support groups and the like do not take place for an indefinite period of time, or only to a very limited extent. | 23 | 64  65  66  67  68 |
| Tension between work and childcare, insecurity at work | 24 | 69 |
| Constant inquiries in the private sphere | 25 | 70 |
| Daughter with cystic fibrosis, fear of infecting her through contact with COVID patients | 26 | 71 |
| Death of close relatives | 27 | 72 |
| Restructuring in the daily routine of the clinic, wearing of masks and thus restrictions in patient communication, decision about COVID testing, which unfortunately was often rejected. In some cases, the clinic management was also overtaxed, which led to uncertainty and a lack of a clear line. | 28 | 73  74  75  76 |
| Uncertainty in handling, stress for children and family members, handling of potentially endangered patients and potentially ill patients with Covid19 | 29 | 77  78 |
| Especially risk to children's well-being in the case of a psychologically stressed mother. | 30 | 79 |

| **Please briefly describe how you solved the challenges.** |  |  |
| --- | --- | --- |
| Answer | Number | Line |
| Own research, consultation with superiors - self-sewn MNS - talk, talk, talk | 31 | 80  81 |
| hardly, by own initiative something is possible in the own surrounding field in the small - I do not carry the socialization to the "social distance" with | 32 | 82  83 |
| endure, meet few people, be transparent in dealing with people, educate patients | 33 | 84  85 |
| Reduction of working hours for me and my partner, so that my daughter does not have to be in emergency care, relatively strict self-protection measures. | 34 | 86  87 |
| Clarification or no solution | 35 | 88 |
| exchange with colleagues at work, video telephony with friends and family | 36 | 89 |
| the children were well taken care of, so I could continue my work | 37 | 90 |
| The problems cannot be solved, or they increasingly show already existing structural problems. | 38 | 91  92 |
| There is no solution. I felt like I was on the verge of a nervous breakdown and now I'm on vacation and trying to distract myself. | 39 | 93  94 |
| Conversations | 40 | 95 |
| generous sick leave, for myself as usual, keep going through.... | 41 | 96 |
| good organization, child had to stay at home alone in part | 42 | 97 |
| I did the measures as recommended despite taunts. But the environment was still a huge stressor, as my family is the most vulnerable because of me. | 43 | 98  99  100 |
| In conversation, after prior information | 44 | 101 |
| In cooperation with my boss, first a detailed conversation with the person concerned, release from the obligation of confidentiality made it possible to consult with the mother's psychiatrist on the phone, Youth Welfare Office could be reached the following day (there is no emergency number outside regular opening hours!!!) and immediately drove to the home visit. | 45 | 102  103  104  105  106 |
| informed, read, talked, talked on the phone, walked | 46 | 107 |
| Contacts minimized | 47 | 108 |
| read, talk, read, talk to friends on the phone, make the best of it | 48 | 109 |
| With exhibition of au and listen | 49 | 110 |
| With the hibweis on my well-deserved closing time , but still contact person for very close friends and family as well as pre-sick friends remained | 50 | 111  112  113 |
| With explanations to family and friends or distance kept | 51 | 114 |
| Together with my husband | 52 | 115 |
| With a lot of calm and time for each individual patient, trying to request more, carefully considering who is tested and referred to Corona centers when material was all | 53 | 116  117  118 |
| After a calm, informative discussion, I acted in the spirit of collaborative decision-making. In most cases, I gave in to the patient's wishes and took the smear test. | 54 | 119  120  121 |
| Open communication and delegation of decisions regarding the scope of contact to the risk group in order to ease the burden on the conscience. | 55 | 122  123 |
| Qualification for emergency care | 56 | 124 |
| Regular meetings and updates regarding current situation and of course exchange with colleagues | 57 | 125  126 |
| Consultation with the boss, gathering information, ensuring childcare. | 58 | 127 |
| Structuring the day Consistent time management through less is more | 59 | 128 |
| Attempt to protect patients and staff with improvised protective equipment Complete reorganization of practice procedures There is not much I can personally do about the isolation in the nursing home | 60 | 129  130  131 |
| a lot of calm and composure Humor is when you laugh anyway a lot of structure | 61 | 132 |
| Total | 62 | 133 |

**Everyone reacts differently to such a pandemic, with different perceptions and ideas. Please briefly describe why you are concerned or not concerned about contracting COVID-19 yourself.**

| Answer | Number | Line |
| --- | --- | --- |
| - Above all, I am worried about infecting or endangering my children, my husband or our parents and my grandmother  - I am also worried that I could infect our patients - especially if I were to spread the disease myself and cause serious illnesses  - I am also worried that the entire practice would have to be quarantined if I were to fall ill  - I am also afraid of claims for damages if I were to start a chain of infections. | 63 | 134  135  136  137  138  139  140 |
| Currently, the situation is well controlled as far as can be judged, everyday measures have been taken and the acceptance of the population is enormous. | 64 | 141  142 |
| As a healthy, young person, my risk for a severe course of the disease is very manageable, so that I am prepared to work on and with COVID patients while observing all protective measures (PSA, isolation of COVID patients). | 65 | 143  144  145  146 |
| Due to the lack of protective measures, initially inconsistent and spongy recommendations. | 66 | 147  148 |
| With protected patient handling and sufficient hygiene measures, I feel fairly safe. It will become more difficult if the existing contact prohibitions are lifted more and more. | 67 | 149  150  151 |
| Professionally, only limited testing is desired. | 68 | 152  153 |
| Covid 19 was never very virulent and in Germany the "wave" was already over by the end of March. I am very worried why because of virtually nothing such massive health-destructive measures have been carried out. And why you again not and again not terminated. | 69 | 154  155  156  157 |
| Since I do not have any chronic diseases, I assume that I will survive an infection with Covid-19 well. The same goes for my family members. | 70 | 158  159  160  161 |
| The current figures show that the risk of infection is low at the moment, so I am not worried at the moment. | 71 | 162  163 |
| Own health and health of the family | 72 | 164 |
| On the one hand, I avoid excessive contact and wear protective equipment in the clinic, and an infection is usually mild. On the other hand, it can affect anyone and the risk of infection cannot be completely reduced. | 73 | 165  166  167 |
| it is not clear how severe the course would be for me and how much I would infect my family and they would suffer from it | 74 | 168  169 |
| low number of cases, enough protective equipment in the practice | 75 | 170 |
| Hold the reproduction factor for very small, find it medially too overestimated, hygiene measures protect | 76 | 171  172 |
| I am a young healthy person and worry little about infecting myself, or rather I think that it could have already happened, but I would certainly survive the disease as such well.  But the organizational hurdles and also the uncertainty of how to behave if you often (and increasingly more often since the relaxations) think you might be infected because a smear result is open after hours and how to organize a quarantine and all the trimmings - that worries me more.  Can I send the children to daycare with a clear conscience if there is a suspected case? Actually, no, but I can't leave them at home all the time either.  I am also worried about the possibility of infecting relatives or friends who may be in the risk group. | 77 | 173  174  175  176  177  178  179  180  181  182  183 |
| I think the pandemic can be controlled. | 78 | 184 |
| I think it is possible to get infected at any time, but I am optimistic that I will "get through" it. I am more worried about infecting my family (small children, grandma) | 79 | 185  189 |
| I don't think that Covid-19 can be dangerous for myself (as a young immunocompetent person). However, if I knew that I was the carrier, I would be even stricter in observing the hygiene rules and keeping the distance. | 80 | 190  191  192 |
| I am not afraid of infection for myself...but to infect my child, my close relatives and my patients. I am young and have no previous illnesses. Since this is still a new thing, it is not possible to estimate how it will affect pregnant women or their newborns. Also the late consequences are not yet foreseeable. | 81 | 193  194  195  196  197 |
| I am not worried about COVID-19, because the risk for a severe course is very low for me and my family. However, I have high-risk patients in my extended family, and unfortunately I am not allowed to see them now. | 82 | 198  199  200 |
| I am not worried because I think I can cope relatively well with an infection. | 83 | 201  202 |
| I am not worried. I take appropriate hygiene measures, and the infection rate is very low, so the probability of getting infected is low. | 84 | 203  204  205 |
| I am not very worried about myself, because the viral infection with SARS-CoV2 in young patients is usually symptomatic and one can protect oneself well with PSA. Compared to other infections I have had to deal with as an inpatient (MRGN4, Pseudomonas, Acenetobacter), CoV2 is rather harmless. | 85 | 206  207  208  209  210 |
| I'm not worried about myself, because I don't have a history of the disease and I think I can get through it without complications. | 86 | 211  212 |
| I do not expect a severe course for myself. | 87 | 213 |
| I also always wear a mask at work and remember to keep my hands hygienic. In this way, I hope not to infect my family. | 88 | 214  215 |
| At work we try to take the best possible protective measures, as far as possible. I am more worried about my parents/grandparents. | 89 | 216  217 |
| At work and in the family, better control is possible. In the private environment, it is difficult to know whether others will also behave considerately. | 90 | 218  219 |
| No fear of the disease itself, but fear of the consequences (quarantine, infection family especially risk group of the family). | 91 | 220  221 |
| no therapy, no vaccination, different information in the media, theoretically could hit everyone, in the neighboring countries also doctors and PP die - one cannot protect oneself properly and permanently | 92 | 223  224  225 |
| No pre-existing diseases, good distance protection, good own infection protection | 93 | 226  227 |
| May it spread faster, but there is no evidence that the Covid19 virus is more dangerous than other viruses. Especially in Germany! | 94 | 228  229 |
| At the moment the situation has eased a lot. few cases, only single positive cases in our practice, good courses. Good working- patients register, separate entrance, separate treatment room. | 95 | 230  231  232 |
| Of course there is an objective risk to get infected with COVID-19. Whether I worry about it or not doesn't change anything. | 96 | 233  234 |
| Not because of me, but because of my daughter, I am very worried to some extent, but on the other hand, I currently assess the risk of contact as very low. In our practice, there has not been a single positive result in the infectious phase, only a few patients who have been through the infection. | 97 | 235  236  237  238 |
| physically healthy, strong immune system, no risk factors | 98 | 239  240 |
| Do not see myself as a risk person, am used to working in "infectious situations" (e.g. influenza patients in hospital) | 99 | 241  242 |
| Worried about infecting myself through the children, as they cannot always keep the distance rules and have to go to kindergarten so that I can go to work. | 100 | 243  244  245 |
| I am worried that the protective measures in our practice were low and were only observed at the insistence of my boss. I am worried that I might infect the very old and chronically ill.  Overall, I am not very worried because the mortality rate of the disease should not be so high due to the high number of unreported cases. | 101 | 246  247  248  249  250 |
| I am mainly worried about an asymptomatic or low-symptomatic infection.  And then less about myself, but about the transmission of the infection. | 102 | 251  252  253 |
| unpredictable outcome, no therapy, I want to live with my family | 103 | 254 |
| We have adapted the consultation hours, we have prepared a lot of information materials for the patients. The patients with infection are taken care of in the infection consultation hour.  I pay attention to hygienic measures and a distance to my patient if it is possible. In addition, there are currently few infected people in our county or village. Also the conversations with my boss have managed many facts, we keep ourselves daily on the "update" and if repeatedly things are heard, a "certain routine/habit" arises. E.g. I watch the webinars with Dr. XXX. Despite the optimism, of course I have respect for CoViD-19 and am "vigilant" :) | 104 | 255  256  257  258  259  260  261  262  263 |

**What other measures have you implemented that have not yet been mentioned?**

| Answer | Number | Line |
| --- | --- | --- |
| - unconditional advance notification by telephone in case of suspected infection - patient information on practice door | 105 | 264  265 |
| Distance rules, also among each other, adhesive tape on the floor, patients get appointments also for picking up, have to wait in the house, only very few come into the practice at all, ALL have to call in advance, triage, we no longer cook together but order, so that no one has to go buying | 106 | 266  267  268  269 |
| all have to register, are not allowed to enter the practice, at the entrance door there is a window with acrylic pane - there patients have to tell the PP what they want, mailbox slot is used to exchange prescriptions, AUs.... There are also pick-up times so that nothing jams (in the hallway), strips on the floor where you have to go and where the chair is for patient | 107 | 270  271  272  273  274 |
| Maintaining sufficient distance, enabled; provided diverse patient education about infection and self-protection. | 108 | 275  276 |
| Measures I have implemented myself are query of risk factors and documentation (was told not to do this), sitting with distance from table, exam with mask/gloves; no more manual medicine techniques, extra gowns; Practice installed mouth guards only 1 week ago, plexiglass 2 weeks ago. Patient load has remained the same, even minor inquiries. Patients are encouraged to come, even if they themselves say they don't need anything. | 109 | 277  278  279  280  281  282 |
| Distance, no handshaking, opening of door by staff when saying goodbye. | 110 | 283 |
| Performing self-swabs in patients' cars outside the office, supervised by a physician colleague. | 11 | 284  285 |
| A separate waiting room was cleared for patients with symptoms of infection. Direct selection at the door of the practice. Temporarily, bills were also available at the counter. | 112 | 286  287  288 |
| Home office is probably not possible in outpatient care. (I would not call telemedicine HO, because it is done from the practice). | 113 | 290  291 |
| In the hospital: Infection ward for suspected corona cases | 114 | 292 |
| Infection tent in front of practice -> no infection patients in practice | 115 | 293 |
| none | 116 | 294 |
| Pediatric practice: toys put away, seating arrangement in waiting room, patients should wear masks if possible (for children 6 years and older) | 117 | 295  296 |
| Only one patient stands in front of the counter/reception, patients are seen sooner due to clocking, laboratory evaluations are discussed more often by telephone. | 118 | 297  298 |
| Patients are signed out and transferred Introduction of video consultation hours Patients are asked to wear masks | 119 | 299  300 |
| Patients are required to wear masks and are told to call ahead if they feel ill. Single entry into the office. Max number of patients time equal in the practice was adjusted. Chairs placed apart | 120 | 301  302  303 |
| Patienten werden rausgeschickt, wenn zu viele auf einmal in der Praxis sind, es wird verscuht, die Patienten etwas zu separieren. | 121 | 304  305 |
| Separation of acute consultations and scheduled routine appointments (check-ups for children), placement in different waiting areas, escorting patients max. 1 person. | 122 | 306  307  308 |
| We put out educational sheets on strengthening the immune system and education that Corona is not a problem. | 123 | 309  310 |
| We have structured the procedures so that as few people as possible come into contact with each other and the doctors no longer work together so that, if possible, one always remains operational. The rest of the practice staff has as little patient contact as possible. | 124 | 311  312  313  314 |
| We have moved appointments apart, the waiting room is constantly aired out/chairs have been removed, we have also brought forward the appointments of our DMP patients under the aspect of few infections in order to then create time resources for an increased number of infections. | 125 | 315  316  317  318 |

**What measures are proving effective/would you like to maintain after "normalization"? Please briefly justify your answer.**

| Answer | Number | Line |
| --- | --- | --- |
| - | 126 | 319 |
| - Pre-registration of all patients by telephone in order to structure the consultation well and ensure spacing | 127 | 320  321 |
| Pick up times for documents for patients for less flow in the office, Clear appointments with as few patients in the office as possible. | 128 | 322  323 |
| Keeping distance, no handshaking | 129 | 324 |
| Acrylic screen at reception, infectious disease consultations, mouth guard for infectious diseases. | 130 | 325  326 |
| Refrain from or severely limit visits from relatives. Brings much more calm to the working day | 131 | 327  328 |
| The work has become more pleasant overall, as the number of patients has decreased. Presentations are currently being made more and more for "valid" health reasons. | 132 | 329  330  331 |
| Avoiding handshaking in the doctor's office.  The separation of acute infection patients from the rest of the patients, for example through an extra waiting room | 133 | 332  333  334 |
| The adjustment of office hours and this clear structuring. | 134 | 335 |
| Protective measures with MNS for infectious diseases must be maintained permanently. In my experience, many older doctors, some of whom are high-risk patients themselves, unfortunately do not do this, or do it too little.  I know practices that did not even use FFP2/3 masks before the pandemic. I don't know whether this was due to ignorance about viral diseases or economic considerations. | 135 | 336  337  338  339  340  341 |
| A critical attitude towards vaccinatio | 136 | 342 |
| one person in front of the counter should continue | 137 | 343 |
| Entering the practice individually - although probably not feasible this is a great help for the staff at the registration desk. | 138 | 344  345 |
| Accurate office hours without everyone coming in when they want. | 139 | 346 |
| Disinfect hands | 140 | 347 |
| Hand disinfection should be available everywhere in public areas | 141 | 348 |
| none | 142 | 349 |
| None in particular; current measures are carried out in this way in principle, even in infectious waves | 143 | 350  351 |
| None unless absolutely necessary. | 144 | 352 |
| Clarify more by phone, better triaging who should come when, not all at the same time at 8 a.m. in practice | 145 | 353  354 |
| Wear mask for throat swab | 146 | 355 |
| Plexiglas, wear mask | 147 | 356 |
| Plexiglas pane to protect nurses | 148 | 357 |
| Plexiglas pane-protects staff from infections at all times, spacing rules-protects from all infections and provides discretion | 149 | 358  359 |
| Conversion of consultation hours Introduction of infection consultation hours | 150 | 360 |
| Video consultation acrylic glass at the registration desk | 151 | 361 |

**What other measures would be useful in your eyes?**

| Answer | Number | Line |
| --- | --- | --- |
| - | 152 | 362 |
| I also find the Plexiglas pane at the counter useful, especially during influenza. Maintaining the current mutual consideration and distance (especially in case of infection). | 153 | 363  364  365 |
| A coronavirus rapid test must soon be available and widely applicable, as it already exists in many emergency rooms for influenza and rhinoviruses. | 154 | 367  368 |
| for all antibody testing as a health insurance benefit | 155 | 369 |
| Separate consultation hours for infections | 156 | 370 |
| Separate office hours for routine visits by chronic patients so that they do not come into contact with acute patients, if possible. | 157 | 371  372 |
| Wear gloves more often | 158 | 373 |
| Isolate high-risk group, normal movement/permitting contamination of immunocompetent patients | 159 | 374  375 |
| Mass testing of normal population Safe antibody testing, rapid normalization for industry | 160 | 376  377 |
| Mouth guards for patients with infectious signs and separate office hours for them Maintain telephone consultations on a smaller scale than now (e.g., for commuters, evaluations, etc.) | 161 | 378  379  380 |
| Official requirement that employees be tested regularly (even without symptoms) - with a clear announcement of who will pay for it | 162 | 381  382 |
| Opening of kindergarten facilities and schools! | 163 | 383 |
| Provide staff with sufficient protective clothing at all times. | 164 | 384 |
| Regular testing of staff | 165 | 385 |
| Regular testing for the virus among staff of health care homes | 166 | 386 |
| telefonische Krankschreibung für maximal eine Woche, vermehrt Onlinezugriff für einfache Rezepte/ Krankschreibung bei unkomplizierte AWI. Warum sollte sich jeder mit einem grippalen Infekt ins Wartezimmer setzen und alle dort anstecken, wenn er 7 Tage im Bett besser aufgehoben wäre?? | 167 | 387  388  389 |
| Training for pandemic situations (as already recommended several times by the civil protection and not implemented), better preparations, set up infection departments with special wards in hospitals. | 168 | 390  391  392 |
| Be more vigilant with politics. | 169 | 393 |

**Do you have any other comments on this topic?**

| Answer | Number | Line |
| --- | --- | --- |
| Working in an orthopedic group practice as a physician in training. | 170 | 394 |
| A rapid coronavirus test must soon be available and widely applicable, as it has long been in many emergency rooms for influenza and rhinoviruses. | 171 | 395  396 |
| It is an absolute indictment that outpatient providers in particular are still provided with almost no protective material! It is an absurdity that there is no obligation for patients to wear masks when visiting medical facilities. It is unacceptable that there are no clear regulations for covering the costs of swabs for contacts from the medical field (without symptoms) and that testing is regulated based on the availability of resources and not according to clear medical indications! | 172 | 397  398  399  400  401  402  403 |
| I find an unreflective publication of numbers without marking upper limits or making comparisons to other infections (like influenza) dangerous, because it puts the population in panic. This panic and uncertainty is the real challenge. | 173 | 405  406  407  408 |
| I, as a physician in training, do not feel taken seriously or protected in my practice. | 174 | 409 |
| No | 175 | 410 |
| Transparency is very desirable, but scare tactics are not helpful. I would like to see better, more uniform concepts that would have better protected the safety of the staff. | 176 | 411  412  413 |
| The handling is so different from country to country, state to state, city to city .... this federal system is out of place here! About other topics is no longer reported - this can not be! For example the changed fee order since 1.March - economic total damage!!! Nursing home residents deteriorate due to being alone and die Corona negative, but alone.  Domestic violence increasing drastically!!! Children have no lobby. | 177 | 414  415  416  417  418  419 |
| much more than Corona in itself , unsettle the working conditions and the e.g. announcement of short-time work and how to deal with it in my current case was now but not made use of it, but it was clearly in the room.... | 178 | 420  421  422 |
| Too much information unsettles the people and makes them afraid, the constant medial Beschallung, particularly with humans, who do not have alternative possibilities, for example only TV and radio, strengthens this fear | 179 | 423  424  425 |

Appendix D - Code system of qualitative content analysis according to Mayring

Stress factor: fear of infection (especially of the family)

Need: communication and support

Stress factor: lack of protective equipment

Stress factor: increased need for patient counseling

Most frequent quantity

| **Code** | **n** |
| --- | --- |
| **1 Stress factors within the personal environment** | 56 |
| 1.1 Burden of social differentiation | 5 |
| 1.2 Fear of psychological effects - family | 2 |
| 1.3 Fear of infection - family | 23 |
| 1.4 Fear of own infection | 6 |
| 1.5 Sense of insecurity regarding the (private) future | 3 |
| 1.6 Difficulties in organizing everyday life | 5 |
| 1.7 Behavioral insecurities | 3 |
| 1.8 Political/media situation as a stress factor | 9 |
| **2 Stress factors in the work environment** | 48 |
| 2.1 Fear of patient infection and consequences | 3 |
| 2.2 Collegial disagreements | 3 |
| 2.3 Increased need for consultation | 23 |
| 2.3.1 Uncontrollable patient behavior | 6 |
| 2.3.2 Increased effort per patient | 8 |
| 2.3.3 Uncertainty in patient care | 9 |
| 2.4 lack of protective equipment | 7 |
| 2.5 Too few or contradictory (protective) instructions for behavior on the job | 9 |
| 2.6 Fear of the future - profession | 3 |
| **3 High stress level / excessive demands** | 1 |
| **4 Aspects that trigger a sense of security** | 33 |
| 4.1 safe health condition | 15 |
| 4.2 Sense of controllability | 18 |
| **5 General feeling of insecurity** | 22 |
| **6 Solution approaches for coping with the stress factors** | 55 |
| 6.1 Strict organization | 4 |
| 6.2 Childcare | 2 |
| 6.3 Self-protection / self-care | 2 |
| 6.4 Work time management | 6 |
| 6.5 Optimization Patient Care | 8 |
| 6.6 Compliance with the rules of conduct | 6 |
| 6.7 none / little possible | 5 |
| 6.8 Expanding support | 15 |
| 6.8.1 Communication / exchange | 10 |
| 6.8.2 Help from trainers/colleagues | 5 |
| 6.9 Production of own protective equipment | 2 |
| 6.11 Independent reconnaissance | 5 |
| **7 Other measures applied** | 40 |
| 7.1 Patient mask | 3 |
| 7.2 Video consultation | 1 |
| 7.3 Communication with laboratories | 1 |
| 7.4 Infectious disease waiting room | 4 |
| 7.5 Self-swabs for patients | 1 |
| 7.6 Reduction of contact points | 2 |
| 7.7 Query of risk factors | 1 |
| 7.8 Controlled distance compliance | 7 |
| 7.9 Increased patient education | 1 |
| 7.9.1 Information on practice door | 2 |
| 7.10 Patient admission strongly controlled | 6 |
| 7.11 strongly controlled collection | 3 |
| 7.12 Reduction of collegial meetings | 2 |
| 7.13 Distance by floor markings | 2 |
| 7.14 Pre-registration by telephone | 4 |
| **8 proven measures** | 32 |
| 8.1 Video consultation | 1 |
| 8.2 none | 3 |
| 8.3 Hand disinfection | 2 |
| 8.4 Individual entry into the registration area | 2 |
| 8.5 Waiting room distribution | 1 |
| 8.6 Regulating the number of visitors from relatives / keeping them under control | 1 |
| 8.7 Masks for infections | 4 |
| 8.8 Separate consultation hours for infections | 3 |
| 8.9 Acrylic disc at reception | 5 |
| 8.10 Pre-registration by telephone | 2 |
| 8.11 Regulated pick-up times | 1 |
| 8.12 Appointment regulation for distance granting | 4 |
| 8.13 Keeping touch points low by keeping distance | 3 |
| **9 Measures desired but not implemented** | 20 |
| 9.1 Training and preparation for pandemics | 1 |
| 9.2 Prescriptions and sick notes online / by telephone | 1 |
| 9.3 Infection departments in hospitals | 1 |
| 9.4 Constantly sufficient protective clothing | 1 |
| 9.5 Employee testing | 3 |
| 9.6 Telephone consultation | 1 |
| 9.7 Masks | 1 |
| 9.8 Separation regulations per risk group vs. immunocompetent groups | 1 |
| 9.9 Gloves | 1 |
| 9.10 Infection consultation | 3 |
| 9.11 Antibody tests | 2 |
| 9.12 Rapid test for Corona | 2 |
| 9.13 Retention of spacing regulations | 1 |
| 9.14 Plexiglas pane/acrylic pane | 1 |
